# Review of advanced research on swine *Actinobacillus pleuropneumoniae* vaccine development strategy

**DOI:** 10.3389/fimmu.2025.1645610

**Published:** 2025-09-16

**Authors:** Adehanom Baraki Tesfaye, Rui Han, Zhengyu Tao, Liuchao You, Jiayao Zhu, Pengcheng Gao, Lei Fu, Yuefeng Chu

**Affiliations:** ^1^ State Key Laboratory for Animal Disease Control and Prevention, College of Veterinary Medicine, Lanzhou University, Lanzhou Veterinary Research Institute, Chinese Academy of Agricultural Sciences, Lanzhou, China; ^2^ Gansu Province Research Center for Basic Disciplines of Pathogen Biology, Lanzhou, China; ^3^ Animal Health Research Team, Tigray Agricultural Research Institute, Mekelle, Ethiopia

**Keywords:** *Actinobacillus pleuropneumonia*, vaccine development, toxins, virulencefactors, immune protection

## Abstract

*Actinobacillus pleuropneumoniae* (App) infection is a major respiratory disease that causes severe economic losses. It is highly infectious and exhibits multiple serotypes, which complicates prevention and control. This review discusses the new-generation vaccine development strategies and the role of virulence factors—such as App toxins, capsular polysaccharide (CPS), lipopolysaccharide (LPS), and outer membrane proteins (OMPs)—in vaccine design. Traditional vaccines offer limited cross-protection, whereas live attenuated vaccines, subunit vaccines, and toxin-based vaccines show promising improvements in efficacy and safety. Current and near-generation subunit and toxin vaccines mainly focus on conserved antigens, incorporating App toxins, OMPs ApfA, and GALT, which significantly enhance cross-protection and safety. Other approaches, including DNA vaccines and combined multivalent vaccines targeting highly prevalent App serotypes and integrating antigens from other pathogens, represent a modern strategy aimed at enhancing cross-serotype protection, minimizing side effects, and enabling differentiating infected from vaccinated animal (DIVA) capability.

## 1 Introduction

*Actinobacillus pleuropneumoniae* is a gram-negative, nonmotile, naturally transformable, facultative anaerobic bacterium with a coccobacillary morphology. The species is classified into two biovars based on nicotinamide adenine dinucleotide (NAD) requirements: biovar 1 strains require NAD for growth, whereas biovar 2 strains do not. Based on cap loci, 19 known serovars have been identified (1), and these serovars can be distinguished from one another using a high-resolution melting assay (2). Focusing on serotype-specific virulence traits, such as lipopolysaccharide (LPS), capsular polysaccharide (CPS), and Repeats in Toxin (RTX) antigenic regions, full-genome profiling has yielded 26 complete circular genomes of *A. pleuropneumonia*. These genomes can serve as a foundation for developing diagnostic tools and vaccines, as well as for establishing whole-genome surveillance and epidemiological studies (3).

The disease is primarily transmitted through direct contact and indirectly via contact with infected piglets (4). The weaning stage represents a critical period for disease spread and a key point for eradication and control efforts (5), as farm management practices can influence the dissemination of *A. pleuropneumoniae* (4). Prior to infection, the bacteria can persist in an inactive state within the tonsils, with pigs acting as passive carriers that transmit the pathogen without clear diagnostic signs. Disease manifestations emerge later due to contributing factors such as stress, compromised immunity, or coinfections, the combination of which complicates disease control (6).

Clinical symptoms of the disease vary in severity among serotypes and primarily affect the respiratory system. Understanding the transmission routes informs control strategies, with strict internal and external biosecurity practices being critical for on-farm management. Vaccination has proven effective in preventing outbreaks, reducing clinical signs, and minimizing mortality on affected farms, making it a preferred method for controlling pleuropneumonia and reducing reliance on antimicrobial treatments (6).

*A. pleuropneumonia* disease is a high-impact, devastating condition that imposes a considerable economic burden on swine production. Various control and prevention strategies can be implemented, but adherence to cleaning and vaccination policies has proven economically significant, especially when disease prevalence is high (7). Many commercial vaccines are available, alongside several vaccine candidates developed through intensive research; however, most vaccines in use are whole-cell inactivated bacterins that lack cross-protection among the major serovars (8). The purpose of this review is to examine the virulence factors involved in vaccine development and to discuss the characteristics of second- and third-generation vaccine strategies in comparison with available commercial vaccines, as well as to explore future directions.

## 2 Virulence factors and their role in the vaccine component

Virulence factors of *A. pleuropneumoniae* have been characterized for their roles in host cell adhesion, essential nutrient acquisition, lesion formation, and evasion of host defense mechanisms. Adhesion-related factors include type 4 pili, trimeric autotransporters, outer membrane proteins, lipoproteins, and lipopolysaccharide (LPS), although their specific host receptors remain undefined (9). The bacterium also expresses multiple virulence determinants critical for colonization, immune evasion, and tissue damage, including fimbriae, pili, OMPs, and surface polysaccharides. Lesion induction and nutrient acquisition involve components such as nickel, zinc, and sulfate uptake, as well as the stress response pathway. Factors involved in biofilm formation and persistence include oxidative stress response, urease activity, stress response proteins, antimicrobial resistance peptides, complement system evasion, and exotoxins (10).

The most well-characterized virulence factors of *A. pleuropneumoniae* include Apx toxins Apx (ApxI, ApxII, Apx III, ApxIV), LPS, CPS, proteases (LonA), ureases, iron acquisition systems (Tbp, Hgbp), enzymes for anaerobic respiration (two-component signal transduction systems such as ArcB/ArcA), pilus structures (type IV pilus, Flp pilus), autotransporters (TAA), and biofilm-forming capabilities (9–12). Information on these virulence factors and their roles in immunogenicity is listed in **Table 1**.

**Table 1:** Illustration of multiple virulence factors involved in host cell defense and vaccine potential.

Cellular structure	Virulence factors	Role	Vaccine-related potential	References
Fimbriae/pili	ApfA, Tfp pilus, TadD	Attachment and colonization	Stimulate mucosal and systemic immunity	(9, 10)
Outer membrane	OmlA, TolC, VacJ, TbpA/B, FhuA	Adherence, nutrient uptake	TbpA, TbpB iron acquisition target; TolC secretion and virulence export channel as a competent subunit	(9, 10, 13)
Surface polysaccharide (CPS/LPS)	CPS, LPS (O-antigen)	Immune evasion, complement resistance	Major target of complement activation and opsonization, useful for serotyping and DIVA strategy	(9, 15)
Periplasmic space	Iron (TbpA (Tbp1 or TfbA) and TbpB (Tbp2 or TfbB), TonB, ExbBD, FhuA. Nickel, zinc (znuABC), sulfate (Sbp)	Nutrient acquisition and transport	Blocks iron or zinc uptake; conserved and surface-accessible immune targets	(10, 13, 15)
Cytoplasmic membrane	Proteins involved in secretion, like TolC	Export of toxins, iron uptake	Virulence export	(10, 16)
Cytoplasm	Apx I–IV, DnaK, SodC, urease, relA, LonA, ClpP	Toxin production, stress response, biofilm formation	Strong toxoid candidate and central to subunit and toxoid vaccines, stimulates Th1 and Th2 responses, supporting immune activation	(9, 13)
Biofilm matrix	PGA, ClpP, LonA	Protection and persistence	Enhance long-term immunity through persistence of biofilm	(9, 13)
Host interaction surface	Adh, AasP, Apa	Host tissue damage and adhesion	Act on the complement system	(9, 10, 13)
General components affecting immune evasion	HlyX, FrpB, PotD, pdxS/T, SapA	Stress resistance, antimicrobial evasion	Contribute to intracellular survival, immune evasion, and activation	(9, 10)

The development of characteristic lesions, including edema, inflammation, hemorrhage, and necrosis, involves multiple virulence factors. Adhesion is mediated by Tfp, Actinobacillus pleuropneumoniae fimbrial adhesin A (ApfA), Adh, and Apa2 proteins, facilitating attachment to host cells. Iron uptake is mediated by FhuA, FhuB, FhuC, FhuD, and HgbA proteins. LamB, a porin-associated protein, contributes to antimicrobial resistance. ApxI, ApxII, ApxIII, and ApxIV mediate lesion formation and immune stimulation, serving as targets for lesion development and immune system activation. Virulnece associated chromosome locus J (VacJ) and HtrA also induce tissue damage. Cell integrity and structural stability are maintained by proteins such as outer membrane lipoprotein A (OmlA), PalA, VacJ, and Polyamine-binding protein D (PotD), and these immunogenic factors collectively serve as targets for developing effective vaccines against *A. pleuropneumoniae* serotypes (13).

Adhesins, iron acquisition factors, CPS, LPS, and RTX, along with their known potential as vaccine components, normally facilitate App colonization, evasion of host clearance mechanisms, and damage to host tissues (14). Hence, CPS and LPS serve a dual role, functioning both as diagnostic markers and as immunogenic components of vaccines. Virulence factors such as purified toxins, CPS, LPS, and OMPs act as immunogens with potential cross-reactivity across App serotypes (15).

Extracellular proteins are critically important for both the survival and pathogenicity of pathogens. Among these, TolC, an outer membrane channel component of the type 1 secretion system, plays a crucial role in *A. pleuropneumoniae*. TolC1 facilitates bacterial resistance, is required for the secretion of ApxIIA and ApxIVA-S toxins, and supports maximum colonization and pathogenicity during infection (16). Most serotypes express four Apx types, with ApxIV being conserved and upregulated *in vivo*, making it an excellent candidate for cross-protective immunity (17). Consequently, ApxIIA is a key virulence factor in *A. pleuropneumoniae* and has been investigated as a potential vaccine candidate (18).

Outer membrane vesicles (OMVs) contain highly conserved proteins, such as ApfA and VacJ, which are expressed by all serotypes of *A. pleuropneumoniae* during infection. These proteins, both individually and in combination with other factors, exhibit immunogenicity and represent potential vaccine candidates. However, their use requires careful selection and characterization to avoid potentially harmful effects (19). OMVs also carry multiple immune-reactive virulence factors, including LpoA, OsmY, MomP, and the hypothetical protein MIDG2331_02184, all of which elicit body responses (20).

Multiple OMPs and lipoproteins play critical roles in colonization, pathogenesis, and virulence. Those that act as a key antigen source for vaccine development include Transferrin Binding Protein A (TbpA), Transferrin Binding Protein B (TbpB), putrescine-binding periplasmic protein (PotD2), capsule polysaccharide-protein (CPxD), and OmlA, which is involved in bacterial survival and host interaction. Similarly, type IV fimbrial subunit protein (ApfA) is a highly conserved factor that facilitates host cell attachment, making it a promising subunit vaccine candidate. TbpA and TbpB are not only virulence factors but also depend on *exbBD* genes to utilize transferrin-bound iron, highlighting their importance for bacterial fitness (21–24).

## 3 *In silico* analysis of vaccine targets

Various researchers have emphasized the importance of *in silico* approaches for identifying vaccine and drug targets in diseases with substantial economic impact. Computer-aided design, artificial intelligence, technologies, and integrated bioinformatics and immunoinformatics methods support the selection of immunogenic targets, epitope prediction, vaccine construction, optimization, and evaluation (25), as well as the identification of suitable peptide targets for vaccine development (26). This trend has been observed in contexts such as tuberculosis (27), Zika virus (28), and *Klebsiella* bacterial infections (29).

In *A. pleuropneumoniae*, using available genome sequences and proteomic database resources, potential targets for vaccine development can be identified. Based on this *in silico* analysis, 11 transmembrane proteins (*frdD*, appser9_7010, *cydA*, *cysT*, *dmsC*, appser1_8310, appser1_4570, *lpxK*, APL_1131, appser4_16420, *ftsl*) were identified among 122 essential proteins, all of which are potential vaccine targets due to their predicted antigenicity. Among these, tetraacyldisaccharide 4′-kinase and 3-deoxy-d-manno-octulsonic acid transferase were nominated as vaccine candidates for inclusion in a vaccine against *A. pleuroppneumoniae* (30).

Another *in silico* analysis method was developed based on three principles. First, the conserved nature of the protein in all strains. Second, the predicted subcellular location is cross-protectively predicted by the presence of an N-terminal signal peptide and similarity to secreted outer membrane proteins. Thirdly novelty of selected proteins from other frequency of studies. Accordingly, conserved *in vivo* expressed outer membrane proteins like comL, lolB, lppC, and ompA, while antigenic, cannot individually protect against colonization or infection. Their efficacy requires a combination with other components (31).

## 4 Type of vaccines

### 4.1 Inactivated vaccines

The first commercialized vaccine against swine *A. pleuropneumonia* infection was a first-generation whole-cell bacterin, consisting of heat-killed or formalin-treated bacteria. Inactivated whole-cell vaccines present diverse antigenic determinants to the immune system while avoiding the reversion risks associated with live attenuated vaccines. Bacteria can be cultured under conditions that mimic the host environment during bacterin preparation to enhance the expression of immunogenic and protective antigens (8). Some recent inactivated vaccines and vaccine candidates are listed in **Table 2**.

**Table 2:** List of inactivated vaccines and vaccine candidates.

Antigenic composition description of the vaccine	Vaccine serotype	Preparation methods	Route of administration	Animal model	Outcomes	References
Inactivated Aptovac^®^ vaccine	App serovars 2 and 6 and *P. multocida.*	An inactivated vaccine that uses aluminum hydroxide gel adjuvant	IM	Pig	Commercially available	(37)
C-vaccine (Coglapix^®^)	App strains of serotypes 1 and 2 (toxins ApxI, ApxII, and ApxIII.	Formalin-inactivated and aluminum gel-adjuvanted	IM	Pig	Commercially available	(32)
Neumosuin^®^	App strains serotypes 2, 4, and 5	Inactivated vaccine	IM	Pig	Commercially available	(37)
Porcilis APP^®^	Exotoxins (ApxI, ApxII, ApxIII) and a 42-kDa OMP multiple serotype	Formaldehyde is inactivated, and antigens are suspended in an aqueous adjuvant	IM	Pig	Commercially available	(37)
Serkel PleuroAP^®^	App serovars 1, 2, 3, 4, and 5.	Oil or aluminum-adjuvanted and formalin-preserved	IM	Pig	Commercially available	(37)
Suvaxyn Respifed APP^®^	Inactivated App serovars 1, 5, and 7.	Formalin-inactivated and oil-adjuvanted	IM	Pig	Commercially available	(37)
Ghost vaccine	Serotype 9	Ghosts were produced by expression of the cloned lysis gene E using the *Haemophilus*–*E. coli* shuttle vector, plasmid pAL2, and were grown in TSB medium	IM	Pig	Candidate, protecting pigs against colonization and infection	(35)
AQ6-AP microspheres vaccine	Serotype 1	Formalin-inactivated App serotype 1 (AP-1) antigen	Oral	Pig and mice	High pig survival and reduced lung lesion areas	(36)

A commercial C vaccine (Coglapix ^®^) was evaluated for its concurrent protective efficacy against multiserovar challenges. The vaccine contains whole-cell *A. pleuropneumoniae* serovars 1 and 2 along with ApxI–III toxins, providing protection against serovars 1, 2, 4, 5, 6, 7, 9/11, and 13. It confers strong protection against both homologous and heterologous serovars, as evidenced by a significant reduction in lung lesions, indicating serovar-independent protection (32).

When *A. pleuropneumoniae* is grown under NAD-restricted conditions, its adhesion to alveolar epithelial cells increases, linked to enhanced expression of fimbriae and OMPs. A bacterin vaccine incorporating serotype 10 was evaluated under both NAD-rich and NAD-restricted conditions. Bacteria cultured under NAD restriction and inactivated by UV light exhibited higher *in vivo* adhesion and provided better partial protection, reducing lung lesions (33).

*A. pleuropneumoniae* ghost vaccines outperformed formalin-killed vaccines in limiting lung colonization. Ghost vaccines for serotypes 9 and 2 demonstrated effective prevention of lung colonization, cross-protective potential, and strong antigen recognition patterns (34). Similarly, when comparing a homologous serotype 9 ghost to its formalin-inactivated counterpart, the ghost vaccine—preserving native surface antigens—caused fewer side effects, blocked lung colonization, and elicited distinct antibody titers (35).

Oral vaccination with three doses of the AQ6-AP vaccine, prepared via a cospray drying process using acetate solution and containing formalin-killed bacteria, provided superior protection compared to intramuscular administration of a formalin-killed inactivated App serotype 1 (AP-1) aluminum-adjuvanted vaccine, resulting in improved pig survival, reduced lung lesions, and fewer clinical signs (36).

### 4.2 Live attenuated vaccines

Vaccination with live attenuated bacteria carries inherent risks, including the potential for reversion to full virulence and the possibility of causing disease in immunodeficient animals. *A. pleuropneumoniae* live attenuated vaccines face challenges such as accidental administration of insufficiently weakened pathogens and the risk of reversion to a pathogenic state. However, a key advantage of attenuated live vaccines for porcine pleuropneumonia is their ability to mimic natural infection. Pigs that survive natural exposure exhibit complete protection against homologous strains and partial cross-protection against heterologous serotypes of *A. pleuropneumonia* (8, 37). Some of the recent live attenuated vaccines and vaccine candidates are listed in **Table 3**.

**Table 3:** List of attenuated vaccines and vaccine candidates.

Antigenic composition description of the vaccine	Vaccine serotype	Preparation methods	Route of administration	Animal model	Protection outcomes	References
Live attenuated serotype 1A riboflavin mutant vaccine	Serotype 1	Live vaccines were grown in Heart Infusion Broth (HIB), harvested through centrifugation, and washed once in PBS	IM	Pig	Protection against mortality and reduces lung lesions and clinical syndromes	(38)
Attenuated *A. pleuropneumoniae* double-deletion mutant S-8ΔclpP/apxIIC	Serovars 7, 1, and 5b	Internal fragment of the *apxIIC* gene was used to introduce the apxIIC mutation into the S-8△clpP mutant	IM	Pig	Conferred efficient protection against homologous or heterologous serovar infection	(39)
Double mutant ΔapxIBD Δpnp forms of *A. pleuropneumoniae* serotypes 1 and 5	Serotypes 1 and 5	*apxIBD* gene deletion was made by trans-conjugation and sucrose counterselection	Intraperitoneal	Mice	Only APP1ΔapxIBDΔpnp offered 75% protection against a homologous challenge	(40)
DapxIC/DapxIIC double mutant of *A. pleuropneumoniae* serovar 1 (SLW03)	Serotype 1	*ApxIC* and *apxIIC* genes were detected by the sucrose counterselection method	Intranasal	Pig	Provide complete protection upon homologous (serovar 1) and heterologous (serovar 9) challenge	(41)
Live attenuated App triple deletion mutant apxIC/apxIIC/apxIV/orf1 (SLW05)	Serotype 1	The *orf1* gene was amplified, cloned, and cut using a single-step trans-conjugation procedure	Intratreachery	Pig	RF1 has made a significant contribution to the development of ApxIVA toxicity. The vaccine provided protection against serovars 1 and 3	(42)
Double mutant *ureC* and *ApxIIA* genes of the *A. pleuropneumoniae* serotype 2 vaccine	Serotype 2	Deletion was performed by homologous recombination and counterselection	Aerosol	Pig	Protects from homologous challenges and aids in serological differentiation of immunized versus vaccinated animals (DIVA)	(44)
Sixfold mutant strain *A. pleuropneumoniae* ΔapxIIA ΔureCΔdmsAΔhybBΔaspAΔfur mutant of serotype 2	Serotype 2	A sixfold mutant strain was constructed by trans-conjugation of the plasmid pFUR702 into the fivefold mutant	Aerosol	Pig	Protection from clinical symptoms upon heterologous infection, serovar 9, and serological discrimination of immunized and infected herds	(45)
znuA mutant strain of the serotype 1 strain SLW01 vaccine	Serotype 1	The *znuA* gene was amplified, and trans-conjugation was performed using plasmid pEDznuA, which was introduced into SLW01 via single-step trans-conjugation	Intratracheal	Pig	Provide 80% and 100% protection against homologous (serovar 1) and heterologous (serovar 7) challenges	(3)
*ApxIIC* gene mutant serovar 7 (HB04C^−^)	Serovar 7	Live cells of nontoxic ApxIIC mutant HB04C^−^ were inoculated on TSB and washed with PBS	Intranasal and intramuscular	Pig	Equal protection to homologous or heterologous (serotype 1) serotypes	(46)
Double mutant strain ΔapxIICΔapxIVA serovar 7 (HB04C^−^)	Serovar 7	Constructed through mutant HB04C^−^ by trans-conjugation and counterselection	Intratracheal	Pig	ApxIV is a critical virulence factor, used as a serological marker for differential diagnosis, and gives good protection as a single mutant	(47)

A live attenuated vaccine was developed by attenuating critical virulence genes. Genetically stable riboflavin auxotrophs were constructed by replacing a segment of the *A. pleuropneumoniae* (App) riboflavin biosynthetic operon (ribGBAH) with a kanamycin resistance cassette. These mutants were shown to be avirulent while still capable of stimulating protective immunity against *A. pleuropneumoniae*. For example, intramuscular vaccination with live attenuated serotype 1A riboflavin mutant, formulated with limiting exogenous riboflavin, provided enhanced protection against an avirulent App challenge. Immunization with these avirulent riboflavin auxotrophs elicited good cross-protection against both homologous and heterologous virulent serotypes (38). Similarly, the S-8△clpP△apxIIC double mutant induced a robust immune response in pigs, characterized by high immunoglobulin (IgG)1/IgG2 levels and elevated production of gamma interferon (IFN-γ), interleukin (IL)-12, and IL-4 production. This mutant provided complete protection against lethal challenge with *A. pleuropneumoniae* serovar 7 or 5a, eliminating lung lesions and reducing bacterial load, positioning it as a promising live attenuated vaccine candidate (39).

Deleting the *apxIBD* gene in *A. pleuropneumoniae* abolishes its hemolytic activity by disrupting the secretion of ApxI- and ApxII-secreting proteins. The resulting APP1ΔapxIBDΔpnp mutant vaccine provides 75% protection against a homologous challenge (App serotype 1) in a mouse model; however, this gene deletion strategy is ineffective against App serotype 5 (40). Similarly, deletion of the *ApxIC* and *ApxIIC* genes in *A. pleuropneumoniae* serovar 1 (SW01) eliminates the secretion of ApxI- and ApxII-activating proteins, generating the attenuated SL03 strain. This strain contains no foreign DNA and secretes inactivated ApxIA and ApxIIA RTX toxins while retaining complete antigenicity. When administered intranasally, the SL03 strain elicits a strong immune response against both homologous (serovar 1) and heterologous (serovar 9) challenges. Therefore, the SLW03 mutant shows potential as a live vaccine candidate capable of providing consistent cross-serovar protection (41).

RTX toxins play a major role in the pathogenesis of APP. The ApxIVA activator (ORF1) is essential for the production of the ApxIVA toxin. Deletion of the *ORF1* gene in serovar 1 generated new strains, including SLW03 (ΔapxICΔapxIIC) and SLW05 (ΔapxICΔapxIICΔorf1). A vaccine formulation containing these two mutant strains, administered intratracheally to pigs, provided effective protection while reducing clinical signs and minimizing lung lesions (42). Similarly, in serovar 1, the *znuA* gene, which is critical for bacterial growth and virulence, produced the ΔznuA mutant from the wild-type SLW01 strain. This mutant conferred 80% protection against a homologous serovar 1 challenge and 100% protection against heterologous serovar 7 challenge in immunized pigs. The ΔznuA strain serovar 1 strain represents a promising live vaccine candidate capable of providing cross-serovar protection following the intratracheal immunization (43).

Attenuation of APP serotype 2 through deletion of highly virulent genes, such as those encoding urease and hemolysin, has yielded promising live attenuated vaccine candidates. A double mutant strain with deletions in ureC (urease) and apxIIA (hemolysin) protects pigs against homologous challenge via aerosol administration and allows differentiation between infected and vaccinated animals (DIVA) (44). Similarly, a live negative marker vaccine from serotype 2 was developed by deleting genes encoding three anaerobic respiration enzymes and the ferric uptake regulator (Fur), creating a highly attenuated six-gene mutant. A single aerosol dose of this mutant conferred significant protection against heterologous serotype 9 infection, an antigenically distinct strain, and allowed clear serological discrimination between vaccinated and infected groups (45).

Another attenuated vaccine derived from App serotype 7 generated via mutation of ApxIIC and ApxIVA toxin genes has demonstrated robust protective efficacy. The ApxIIC mutant strain HB04C (serovar 7) protected mice against App infection, with intranasal and intramuscular administration yielding equivalent efficacy. This strain elicited significant protection against experimental challenges with both homologous (serovar 7) and heterologous (serovar 1) virulent strains, positioning it as a promising vaccine candidate (46). Additionally, an apxIIC/apxIVA double mutant was developed as an effective live marker vaccine, enabling serological differentiation between vaccinated and infected animals (47).

### 4.3 Subunit vaccines and vaccine candidates

Bacterial surface components, including capsule, lipopolysaccharide, and various outer membrane proteins (OMPs), constitute important classes of antigens. Among these, transferrin-binding proteins and heme-binding proteins were the first to be identified as potential vaccine candidates (23). Certain subunit vaccines have entered the commercial market; these vaccines incorporate Apx toxins along with outer membrane proteins or bacterial cells. According to reports, they offer superior cross-protection compared to traditional bacterins (48). For instance, a commercial subunit vaccine formulated with ApxIA, ApxIIA, ApxIIIA, and OMP2 is known as the Porcilis vaccine. This vaccine is recognized for its high degree of protection, significantly decreasing the incidence of pleurisy and pneumonia in pig farms. Additionally, it reduces antimicrobial usage (49). Some more recently developed subunit vaccines and vaccine candidates are listed in **Table 4**.

**Table 4:** List of subunit vaccines and vaccine candidates.

Antigenic composition description of the vaccine	Vaccine serotype	Preparation methods	Route of administration	Animal model	Protection outcomes	References
*A. pleuropneumoniae* (serotypes 2 and 9) in the absence of TbpA, with alkaline phosphatase (Hsp60) detected in cell-free supernatant (CFS) OMs	Serotypes 2 and 9	Antigen preparations from APP serotypes 2 and 9 were grown under iron-restrictive conditions using sodium deoxycholate extraction	IM	Pig	Strong antibody response, immunized pigs showed no or only mild clinical signs	(51)
Recombinant *GalT* gene in *in vivo*-induced antigen of *A. pleuropneumoniae*	Serotype 5b	GALT protein plus adjuvant	SC	Mice	Effective cross-protective antigen against APP serovar 1 MS71 (50%) and APP serovar 5b L20 (75%)	(52)
Lipoproteins (APJL_0922, APJL_1380, and APJL_1976) of serovar 3	Serovar 3	Cloned, expressed immunogenic potential of the lipoproteins was determined in mice by ELISA and immunoblotting	IM	Pig	Potential subunit vaccine candidates provide protection against heterologous serotype 1	(53)
Three proteins (APJL_0126, HbpA, and OmpW) of serovar 3	Serovar 3	Selected through bioinformatics, gene expression, and purification	Intra-tracheal	Pig	High antibody titers and lower clinical signs	(54)
Extracellular vesicles (APP-EVs) from App serotype 5	Serotype 5	Extracted from bacterial culture supernatants through by step-by-step process involving centrifugation, tangential flow filtration	IM	Mice	Stronger cellular immunity than the Coglapix vaccine	(55)
Proteins (rGalT [App2], rAPL_1166 [App 4], and rHflX [App 3] antigens)	Serotypes 2, 3, and 4	Bioinformatics selection, cloned, expressed, and purified	Subcutaneous	Mice	Survival rate 62%, 5%, and 87.5%, partial protection	(56)
ApfA (App7) + (rApxIA, rApxIIA, rApxIIIA, rApxIVA, and rTbpB proteins)	Serotypes 1, 2, 3, and 7	Purified recombinant antigens added in oil adjuvant	IM	Pig	ApfA is a good component added to the vaccine to improve protection	(58)
DIVA subunit vaccine ApxII toxin	Serotypes 1, 2, and 5	Vaccine antigen is prepared using a single-step trans conjugation system; half of these serotypes were washed using a detergent, saline emulsifier	IM	Pig	Complete protection against homologous and heterologous strains and reduced colonization	(50)

A vaccine known for its DIVA capability was developed by extracting OMPs and secreted proteins using a detergent wash method. The vaccine incorporates the highly immunogenic ApxII toxin, present in 13 of the 15 App serotypes, as a DIVA antigen. The *Apx* gene was deleted from a single strain of each of serotypes 1, 2, and 5 using a single-step trans-conjugation system. Equal amounts of the detergent washes from these modified strains were used as the vaccine antigens. After intramuscular immunization, all pigs mounted a robust humoral immune response to the vaccine antigen and showed no positive reaction in an ApxIIA Enzyme linked Immuno sorbery assay (ELISA). In challenge trials, all vaccinated pigs were fully protected from symptoms when exposed to both a homologous strain (App 2) and a heterologous strain (App 9). Moreover, the colonization of the challenge strain was significantly reduced, although it was not completely eliminated. Due to the high level of protection provided by the vaccine, immunized pigs do not develop detectable levels of antibodies to the DIVA antigen in the ELISA. Instead, only a more sensitive Western blotting technique could identify these antibodies, highlighting the difficulties in creating suitable marker vaccines for the livestock industry (50). Similarly, antigens prepared from App2 and App9, which lacked the TbpA protein, induced an antibody response against the serotype 2 challenge, resulting in a reduction in lung lesions (51).

When pigs were vaccinated with a combination of subunit recombinant proteins—namely rApxI, rApxII, rApxIII, and rOMP—they exhibited elevated antibody titers and survival rates, along with reduced lung lesions. This vaccine combination provided more effective cross-protection against both homologous and heterologous challenges from App, specifically serotypes 1 and 2. In comparison, other combinations, such as those including rApxI, rApxII, rApxIII, rApxIV, rApfA, and rOMP, as well as the inactivated App1 vaccine, offered less robust protection (48).

Bioinformatics analysis showed that the *Galactose-1-Phosphate Uridylyltransferase (GALT)* gene is highly conserved across App strains. Animals vaccinated with GALT exhibited effective cross-protective immunity. As a result, GALT has the potential to serve as a vaccine against multiple App serotypes (52).

It has long been recognized that numerous lipoproteins possess immunoprotective properties. In the case of App strain JL03 (serovar), lipoproteins APJL_0922, APJL_1380, and APJL_1976 induced significant humoral immune responses. Moreover, these lipoproteins conferred effective protective immunity against challenges from heterologous and virulent App (App serovar 1), as reported by Cao et al. (53). Similarly, through bioinformatics and experimental identification of surface-associated immunogenic proteins for vaccine formulations, conserved OMPs, lipoproteins, and Apx toxins have been recognized as potential vaccine candidates. Genes (*APJL_0126*, *HbpA*, and *OmpW*) amplified from App serovar 3 (JL03) induced high antibody titers and low clinical scores against both homologous and heterologous challenges. However, these conserved genes need to be combined with Apx toxins to achieve full protective efficacy (54).

Extracellular vehicles (EVs) derived from APP hold promise as vaccine candidates. Compared to the Coglapix vaccine, App-EVs can trigger App-specific Th1, Th17, and cytotoxic T lymphocyte (CTL) responses, and also promote the activation of multifunctional T cells. These properties enable App-EVs to enhance the protective response against App infections (55).

App has numerous identified antigens involved in metabolism, replication, transcription regulation, signal transduction, and other functions. Among these, six *in vivo*-induced tagged proteins are potential vaccine candidates. In a mouse model, three proteins from serotypes 2, 3, and 4—rGalT (App2), rAPL_1166 (App4), and rHflx (App3)—showed a notable survival rate and provided partial protection against App infection (56).

Outer membrane vesicles are significant immunogens with antigenic similarity, making them promising vaccine candidates. Vaccination strategies included administering recombinant proteins of ApfA and VacJ individually or in combination with OMVs. Although the addition of OMVs increased the IgG levels, it did not provide sufficient protection; instead, it led to increased lung lesions, providing evidence that antibody-mediated cytotoxicity in the host immune response may play a crucial role in the development of lesions associated with App infections (57). The type IV fimbrial protein (ApfA), when combined with recombinant antigens in a hexa-antigen combination (rApxIA, rApxIIA, rApxIIIA, rApxIVA, rTbpB, and rApfA), elicited strong immunogenicity and contributed to the development of a valuable subunit vaccine for preventing App infections (58).

### 4.4 Toxin-based vaccines

Apx toxins, members of the RTX toxin family, are secreted and represent major virulence factors of App with strong immunogenicity (8). Four distinct Apx exotoxins exist, each with different functions and virulence levels. ApxI, ApxII, and ApxIII, the most crucial virulence factors, are produced and secreted into culture supernatants, whereas ApxIV is expressed *in vivo* and detected during natural infection (59). Owing to their high immunogenicity, both commercial and experimental vaccines incorporating Apx toxins have been developed. App serotypes 1 and 5 are among the most virulent, producing both ApxI and ApxII toxins (60). Some of the recent toxoid-derived vaccines and vaccine candidates are listed in **Table 5**.

**Table 5:** List of toxoid vaccines and vaccine candidates.

Antigenic composition description of the vaccine	Vaccine serotype	Preparation methods	Route of administration	Animal model	Protection outcomes	References
bivalent fusion l-vaccine (ApxIA and ApxIIA) fragments	Serotype 1	Genes were constructed, ligated, transformed into the same host, harvested, and purified	Intraperitoneal	Mice	Induces specific humoral and cellular immune responses and cross-protection to serotype 2	(61)
Trivalent Apx fusion protein enclosed in outer membrane vesicles (Apx(I-III)r-OMV)	Serotype 1	ApxIAr, ApxIIAr, and ApxIIIAr fusion protein OMVs were prepared and expressed. ClyA-Apxr fusion protein OMVs were used without an oil adjuvant	SC	Mice	Induce specific humoral or cellular immune responses, as well as cross-protective immunity against different serotypes, such as 1 and 7	(62)
*Saccharomyces cerevisiae* expressing Apx toxins (ApxIIA#5 and ApxIA) vaccine	Serotype 2	Surface-displayed ApxIIA#5 was expressed in *S. cerevisiae*, and the full ApxIIA was expressed, fused to yeast, and cloned	Orally	Pig	Showed higher specific IgG and IgA, and lower lesion scores	(65)
Fragment #5 of ApxIIA serotype 2	Serotype 2	Partial fragment #5 of the *ApxIIA* gene was amplified, and the recombinant antigen was produced	Intranasal	Mice	Inhibited bacterial colonization and cross-protected against heterologous serotype (serotype 5)	(67)

The l-vaccine was developed by formulating genes encoding the NA region of the full *ApxIA* gene and the F#5 region of the full *ApxIIA* gene. These genes were amplified by PCR from the genomic DNA of App serovar 1. Initially, the ApxIA and ApxIIA proteins were used for monovalent vaccines; when combined, they formed the bivalent l-vaccine. This vaccine elicits robust humoral and cellular immune responses, providing complete cross-protection against App infection (61).

A trivalent fusion protein composed of ApxI, ApxII, and ApxIII, encapsulated within outer membrane vesicles (Apxr-OMV), induces both humoral and cellular immune responses and reduces histopathological lesions. When used as a novel vaccine, Apxr-OMVs provide cross-protective immunity against infection by App serotypes 1 and 7 in a mouse model (62).

A study compared two major commercial vaccines, both containing conserved Apx toxins, to assess their impact on reducing mortality and clinical lesions. The research found that Coglapix^®^, a commercial pig vaccine featuring App1 and App2 along with ApxI, ApxII, and ApxIII toxins, outperformed Porcillis App^®^, a subunit toxoid vaccine with ApxI, ApxII, and ApxIII toxoids and OMP antigen. Specifically, Coglapix^®^ was more effective in lowering lung lesions, mortality, and the need for antimicrobial treatment in nursery pigs, thereby providing better protection against pulmonary lesions caused by App infection (63).

*A. pleuropneumoniae* possesses a cytoplasmic *N*-*N*-glycosylation system responsible for modifying high molecular weight adhesions by adding glucose residues. The soluble *N*-glycosyltransferase (ngt), encoded in an operon alongside a subsequent glycosyltransferase, was used to develop a glycoconjugate vaccine. In this approach, recombinant *Escherichia coli* expressed a soluble Apx toxin fragment, which was subsequently glycosylated with glucose. This vaccine candidate, combining a toxin fragment with a conserved glycan, offers a new way to generate epitopes critical for both bacterial colonization and disease progression (64).

The Apx toxin of APP was expressed in *Saccharomyces cerevisiae* yeast and evaluated as an oral vaccine. ApxI and surface-displayed ApxII#5, derived from serotype 2 and expressed in *S. cerevisiae*, elicited a robust immune response against serotype 5 challenges. These findings have informed the development of a live oral vaccine for porcine pleuropneumonia, providing an alternative to traditional vaccines (65). Notably, the ApxII toxin is the most commonly expressed among the 15 bacterial serotypes, except for serotypes 4 and 10. A peptide ligand capable of targeting the ligand-conjugated ApxIIA#5 fragment antigen was identified as an effective adjuvant. This adjuvant induced both mucosal and systemic immune responses against a serotype 2 challenge (66), indicating that the ApxIIA fragment #5 contains a crucial epitope for vaccination. Intranasal immunization with fragment #5 not only elicited strong systemic and mucosal immune responses but also inhibited bacterial colonization and prevented tissue damage following a serotype 2 challenge. Moreover, it provided cross-protection against heterologous serotype 5. As a result, this approach is regarded as an efficient method for protective immunization against APP infection (67).

### 4.5 DNA vaccines

DNA vaccines represent a third-generation vaccine strategy and offer several advantages over traditional vaccines, including enhanced safety, thermostability, and ease of production. Their molecular structure provides inherent stability, and the antigens they encode are highly specific. DNA vaccines can elicit both humoral and cellular immune responses and can be manufactured in large quantities with high purity. Even a small amount of plasmid DNA is sufficient to generate a robust immune response (8, 68).

Several reports have reported DNA immunization strategies aimed at controlling App infection, with DNA vaccines encoding structural proteins of Appx exotoxins showing promising results. A divalent DNA vaccine sourced from serotype 1, consisting of pcDNA-apxIApcDNA, apxIA, and pcDNA-apxIIA, when administered intramuscularly, induced a significant humoral immune response. This response included activation of both Th1 and Th2 cells against a lethal serotype 1 challenge, suggesting that this approach could serve as an innovative strategy for preventing App infection (69).

Building on this work, another study focused on the type IV fimbrial protein (ApfA), a highly conserved structural protein across multiple App serotypes (70). A DNA vaccine encoding ApfA (pcDNA-apfA) was developed to evaluate its protective efficacy against App serotype 2 infection. The antibody response elicited by pcDNA-apfA, however, conferred only limited protection, achieving 30% efficacy against the serotype 2 challenge. Despite this modest protection, the vaccine successfully induced an immune response, suggesting its potential as a component of a multivalent DNA vaccine for next-generation strategies to combat APP infection in pigs (71).

### 4.6 Combined vaccines and vaccine candidates

Several vaccine combinations can be used either within the App formulation of different vaccine candidates (72) or by including other bacteria that enhance the efficacy of App-based vaccines. These combinations aim to improve the protection of major respiratory diseases affecting pig production while simultaneously controlling the recurrence of such diseases (73). Some of these combined vaccines and vaccine candidates are listed in **Table 6**.

**Table 6:** List of combined vaccines and vaccine candidates.

Antigenic composition description of the vaccine	Vaccine serotype	Preparation methods	Route of Administration	Animal model	Protection Outcomes	References
(Bac-Sub) vaccine inactivated bacterial cells serovar 1 and (rApxIA, rApxIIA, and rApxIIIA)	Serotypes 1, 3 and 7	APP 1(HB01strain)inactivated(5x10^8^CFU) and mixed with amplified, cloned, and purified recombinant (rApxIA, rApxIIA, and rApxIIIA) 125 µg of each	IM	Pig	provided greater protection, lower lung lesions, and bacterial load	(72)
Multivalent *A. pleuropneumoniae* and Mycoplasma hyopneumoniae vaccine	App Serotype 1, 2, and 5, Mhp strain HID3140	formalin-inactivated bacterins plus (ApxI, ApxII, and ApxIII) were produced, optimized, and purified from App serotypes 1, 2, and 5 plus inactivated Mhp; and purified rP97 surface adhesin	IM	Pig	Promising vaccine for the prevention of two respiratory diseases better than Nisseiken Swine APM Inactivated Vaccine	(73)
multi-disease subunit vaccine A. Pleuropneumoniae and M. hyopneumoniae (Ap97) (Recombinant Chimeric Ap97)	App serotype 2, *M. hyopneumoniae* strain J	Mixed containing a fragment of the apxIIIA with deleted N terminal region and R1 and R2 repeats of *M. hyopneumoniae* P97 adhesin (P97C) formed chimeric protein Ap97 administered in complete Freund's adjuvant.	Sub cutaneous	Pig	Increased IgG and Cytokines response against both diseases challenges	(74)
Multicomponent Combined vaccine (rApxI + rApxII + rApxIII + rApxIVN + rOMP)	Serotypes 1, 2 and 7	Purified proteins consisted of equivalent amounts of rApxI, rApxII, rApxIII, rApxIVN, and rOMP so that 25 gram of each protein in 1 ml of PBS mixed together with 1 ml of Freund's adjuvant	IM	Pig	Higher Ab titer, reduced lung lesion and low bacterial recovery	(75)
Multi-component vaccine inactivatedAPP1 plus(ApxI, ApxII, ApxIV, and OMP)	App serotype 1	ApxI, apxII, apxIV, and OMP genes were expressed and purified then 1 × 10^9^ CFU inactivated App1 and 150 μg of each recombinant protein Water in an oil emulsifier	IM	Pig	provided complete protection and reduced gross lesions by 67%	(76)
Multicomponent recombinant vaccines (rApxI, rApxII, rApxIII and rOMP)	App1 and App2	rApxI, rApxII, rApxIII, and rOMP cloned, expressed, and purified emulsified with mineral oil	Sub cutaneous	mice	Higher Ab and survival rate and no lung lesions observed	(48)
Combined Recombinant tandem epitope-based (RTA) +inactivated APP5b )vaccine	App 5	*rta* gene cloned, transferred and expressed, purified, and formalin-inactivated App 5 was added mixed with aluminum-gel brine adjuvant	Subcutaneous	Mice	strong cross immune protection in mice(100%), lower clinical symptoms	(77)
protein antigens of Propionibacterium acnes(PA-Ssb) for Actinobacillus pleuropneumonia	P. acnes strain S4 a biotype I-B strain	Protein gene from P. acnes S4, a biotype I-B strain which was isolated from human acne lesions, cloned, expressed, and purified mixed with Freund’s adjuvant	Intra peritoneal	Mice	PA-Ssb induces the highest titers of antibody and cross-reactive (ApxIV toxin and *Znu*) against *A. pleuropneumoniae* serotype 1 and 5	(78)
Live attenuated *A. pleuropneumoniae* Triple-Deletion Mutant apxIC apxIIC apxIV-ORF1 (Strain SLW05) against *H. parasuis* vaccine.	App serotype 1	(ΔApxIC, ΔapxIIC ΔapxIV-ORF1) a mutant strain of *A. pleuropneumonia*, (SLW05) grown on TSB media and diluted in PBS as a vaccine	IM	Pig	Induce Th1, IL2, and IFN-gamma finally protect against lethal challenge with virulent *H. parasuis* SH0165 (serovar 5) or MD0322 (serovar 4)	(79)

Vaccines can be formulated in combinations to maximize protection against the wide variety of APP serotypes. One study compared a novel combined vaccine, Bac-sub, with existing commercial vaccines. Bac-sub consisted of inactivated bacterial cells from serovar 1, combined with three recombinant protoxins (rApxIA, rApxIIA, and rApxIIIA), and emulsified with adjuvant Montanide IMS7. The commercial vaccines included a subunit vaccine containing ApxI, ApxII, and ApxIII toxoids along with OMPs, and a trivalent bacterin made from inactivated bacterial cells of serovars 1, 2, and 7. In comparison, the Bac-sub vaccine showed no significant side effects. After booster immunization, it induced higher levels of Apx toxin-specific IgG, IgG1, and IgG2a compared to the commercial trivalent bacterin and subunit vaccines. When challenged with virulent strains of serovars 1, 5, and 7, the Bac-sub vaccine provided super protection, with efficacy rates of 91.76%, 100%, and 100%, respectively. It also resulted in lower lung bacterial loads and fewer lung lesions. These findings indicate that the Bac-sub vaccine is a safe and effective combination vaccine offering robust protection against App infection (72).

To optimize pig production on farms, multivalent vaccines targeting common respiratory diseases, such as porcine pleuropneumonia and *Mycoplasma hyopneumoniae* (Mhp), are worth considering. One such vaccine was developed by combining App bacterins and RTX toxins with Mhp bacterin and adhesion proteins. The App component includes serotypes 1, 2, and 5 bacterins, along with AppxI, AppxII, and AppxIII toxoids, providing protection against virulent App serotypes 1, 2, and 5. The Mhp component consists of inactivated Mhp and recombinant P97 surface adhesion proteins. This new combined vaccine was evaluated against the commercial Nisseiken Swine APM inactivated vaccine. The vaccine not only reduces injection-induced stress in pigs but also outperforms the commercial vaccine in stimulating antibody production, lowering lung lesions, and improving recovery rates. It shows promise as an effective measure for preventing both porcine pleuropneumonia and mycoplasmal pneumonia (73). Similarly, a multidisease subunit chimeric protein vaccine was developed by combining the N-terminal conserved ApxIII toxin (ApxN) of App serotype 2 with the C-terminal conserved region of *M. hyoppneumoniae* P97 adhesin (P97C). Subcutaneous administration increases IgG levels and provides protection against both pathogens (74).

The role of the Apx IV toxin of App in vaccine development was assessed by including it in multicomponent recombinant subunit vaccines. Combining ApxIV with other components (rApxI + rApxII + rApxIII + rApxIVN + rOMP) offered better protection than using rApxIVN alone. Compared to a vaccine formulation of rApxI + rApxII + rApxIII + rApxIVN + rOMP, it resulted in higher antibody levels, milder lung lesions, and lower bacterial recovery when challenged with App serovars 1 and 2 (75). Similarly, adding recombinant ApxIV to a multicomponent vaccine containing ApxI, ApxII, ApxIV, and OMP, together with inactivated App serovar 1, provided full protection and reduced visible lesions after both homologous (serovar 1) and heterologous (serovar 5) challenges (76).

An epitope-based vaccine development was carried out. It involved selecting some trimeric autotransporter adhesion-based B-cell epitopes and constructing a recombinant tandem antigen (RTA). Vaccine trials were conducted using this RTA protein alone and in combination with other formulations. When RTA was combined with activated App 5b, it significantly enhanced the cross-protection to 100% against a heterologous App serotype 1 challenge. It was found that the RTA protein, when combined with a suitable inactivated APP strain, could potentially serve as a candidate vaccine (77).

*Propionibacterium acnes*, a bacterium isolated from the human face, showed immunological cross-reactivity with pig App strains. Six *P. acnes* proteins recognized by App-specific serum were evaluated for vaccine development. In the mouse challenge model, they provided partial protection against App serotypes 1 and or 5 infections. Protection might be partly mediated by small peptide sequences in the *P. acnes* single-standard DNA-binding protein, which cross-react with sequences in the App ApxIV RTX toxin and zinc-binding protein (ZnuA). These findings suggest that *P. acnes* could be a valuable vaccine candidate against App serotypes (78).

*Haemophilus parasuis* and App both belong to the Pasteurellaceae bacteria family and may share cell wall antigenic sites, potentially enabling cross-protection. The attenuated App serovar 1 live vaccine prototype SLW05 (apxIC apxIIC apxIV-ORF1) triple deletion mutant strain not only elicits protective immunity against App but also protects against a lethal challenge from virulent *Haemophilus parasuis* strain SH0165 (serovar 5) and MD0322 (serovar 4). It induces a Th1-type immune response, stimulating IL-2 and IFN-γ production, offering a new approach for developing an attenuated *H. parasuis* vaccine (79).

## 5 Concluding remarks

The disease caused by App is a severe bacterial disease faced by the global pig breeding industry. Its high infectivity and multiserotype characteristics make prevention and control difficult. Currently, vaccines remain the most effective means of controlling the disease, but existing vaccines have obvious limitations. The first-generation vaccines inactivated whole-cell vaccines; although they can induce an immune response, their cross-protection ability among serotypes is limited. Moreover, they rely on large amounts of bacterial antigens, which may cause adverse reactions. Attenuated live vaccines, in which pathogenicity is reduced through gene deletion—such as knocking out virulence genes like *Apx* and *ZnuA*—can induce strong cross-protection, but there is a risk of virulence reversion, and the production process is complex. The third-generation vaccines, such as subunit vaccines, toxin vaccines, and DNA vaccines, which mainly focus on conserved antigens such as Apx toxins, OMPs, and ApfA GALT, significantly enhance cross-protection potential and have higher safety. For instance, subunit vaccine-based ApxI–III toxins, such as commercially available Porcilis APP^®^ and OMV vaccines, have shown better protection effects than traditional vaccines. Combined vaccines, made from multiple components such as App toxins and antigens from other bacteria like mycoplasma, or designed as multipathogen combinations, can prevent and control multiple respiratory diseases simultaneously, reducing the number of immunizations and improving farm-based economic benefits.
